# Hepatitis A in the Eastern Mediterranean Region: a comprehensive review

**DOI:** 10.1080/21645515.2022.2073146

**Published:** 2022-05-26

**Authors:** Selim Badur, Serdar Öztürk, Mohammad AbdelGhany, Mansour Khalaf, Youness Lagoubi, Onur Ozudogru, Kashif Hanif, Debasish Saha

**Affiliations:** a GSK, Istanbul, Turkey; b GSK, Cairo, Egypt; c GSK, Jeddah, Saudi Arabia; d GSK, Casablanca, Morocco; e GSK, Dubai, United Arab Emirates; f GSK, Karachi, Pakistan; g GSK, Wavre, Belgium

**Keywords:** Eastern Mediterranean Region, endemicity, hepatitis A, incidence, seroprevalence

## Abstract

**Introduction:**

With 583 million inhabitants, the Eastern Mediterranean Region (EMR) is a worldwide hub for travel, migration, and food trade. However, there is a scarcity of data on the epidemiology of the hepatitis A virus (HAV).

**Methods:**

The MEDLINE and grey literature were systematically searched for HAV epidemiological data relevant to the EMR region published between 1980 and 2020 in English, French, or Arabic.

**Results:**

Overall, 123 publications were extracted. The proportion of HAV cases among acute viral hepatitis cases was high. HAV seroprevalence rate ranged from 5.7% to 100.0% and it was decreasing over time while the average age at infection increased.

**Conclusion:**

In the EMR, HAV remains a significant cause of acute viral hepatitis. The observed endemicity shift will likely increase disease burden as the population ages. Vaccinating children and adopting sanitary measures are still essential to disease prevention; vaccinating at-risk groups might reduce disease burden even further.

## Plain Language Summary

What is the context?

- Hepatitis A is a viral liver disease caused by the hepatitis A virus.
- It is generally transmitted by ingestion of contaminated food or water or through contact with an infected person.
- Disease severity increases with age. Children under 6 years of age are usually asymptomatic, while adults are the most affected.
- Limited information exists on the number of cases and transmission of hepatitis A in the Eastern Mediterranean region, which includes 21 countries and Palestine, as defined by the World Health Organization.

What is new?

- We performed a literature review to summarize data on hepatitis A disease in the Eastern Mediterranean region over the last 40 years (1980-2020). As information for many countries is scarce or outdated, most of the data is from Egypt, Iran and Saudi Arabia.
- We found that:
  - Hepatitis A virus is the most common cause of acute viral hepatitis.
  - Hepatitis A exposure varied according to the country’s income level.
  - Low- and middle-income countries showed a universal immunity to hepatitis A virus, although this is not the case anymore.

What is the impact?

- Hepatitis A infections have decreased worldwide. Lower exposure to the virus has led to an increase in the susceptible population (including adolescent and adults).
- Hepatitis A vaccination for children and high-risk groups such travelers should be considered in the Eastern Mediterranean region.

## Introduction

Exposure to the hepatitis A virus (HAV) causes viral hepatitis which is characterized by inflammation of the liver. Globally, more than 100 million HAV infections and 30,000–35,000 deaths are reported annually.^1^ HAV is transmitted through the fecal-oral route, entering via the mouth and replicating in the liver.^1^ The ingestion of contaminated food or water, poor sanitation, and contact with an infected individual are the primary sources of infection.^1,2^ Clinically, HAV infection is similar to other types of acute hepatitis, with elevated levels of liver enzymes, dark-colored urine, and the onset of jaundice. It is accompanied by broad symptoms like fatigue, malaise, and abdominal pain.^3^ The severity and outcome of the disease is negatively correlated with the age at infection. Infected children under six years of age are usually asymptomatic (~70% cases), while older children and adults show symptoms of jaundice (~70% cases).^3^ The fatality rate increases with increasing age, from 0.1% (<15 years of age), to 0.3% (15–39 years of age) and 2.1% (≥40 years of age).^4^ Infection due to HAV can be diagnosed by serological testing in the presence of anti-HAV immunoglobulin M (IgM) and immunoglobulin G (IgG).^5^ The presence of IgM antibodies is indicative of a recent HAV infection, while the detection of IgG antibodies suggests previous exposure to HAV or vaccination, as IgG antibodies persist over time and confer lifelong immunity.^3,5^ The measurement of IgG antibodies is an indirect method of measuring seroprevalence, overall and by age, and can be used to assess the endemicity level (*i.e*., the circulation of the HAV) in a given population.^2^

Inactivated and live attenuated hepatitis A vaccines have proven to be immunogenic, well tolerated and safe in the target-vaccine population.^6–8^ The World Health Organization (WHO) recommends the inclusion of hepatitis A immunization into the national immunization schedule for children ≥1 year of age, taking into consideration the incidence of acute HAV cases, the endemicity level (high to moderate), and cost-effectiveness data.^2^ Notwithstanding this recommendation, the WHO states that vaccination should be part of a comprehensive plan for the prevention and control of viral hepatitis, including measures to improve hygiene, sanitation and outbreak control.^2^

Broader access to clean water and sanitation, and improved socio-economic conditions are changing the epidemiology of HAV infection.^9,10^ Due to globalization, rising income, and better infrastructure, low- to middle-income countries are undergoing a shift from high/intermediate to low HAV incidence rates, and high-income countries are now non-endemic to HAV infection.^11^ Importantly, countries reporting low or intermediate HAV endemicity, including those countries in transition from high to low HAV endemicity, are particularly susceptible to recurrent outbreaks of symptomatic disease.^12^

Given this context of evolving HAV epidemiology, the WHO Eastern Mediterranean Region (EMR) deserves attention. The EMR includes 21 member states and Palestine comprising nearly 600 million people.^13^ This region is comprised of middle-income (11) as well as high-income (6) and low-income (5) countries as classified by the World Bank (2017).^14^ In the last decade, EMR countries have documented a significant improvement in their socio-economic conditions. Advances in modern transportation and global accessibility, in particular, have boosted the travel and food industries. However, the EMR has also seen a rise in armed conflict, which has increased the rate of human migration and disease mobility. As a result, the EMR reports the highest global number of people displaced from their home countries.^15^ Refugees displaced from high endemicity countries represent a source of contagion for their new country, especially if their housing is crowded and with poor sanitation and hygiene conditions.

**Figure 1.**
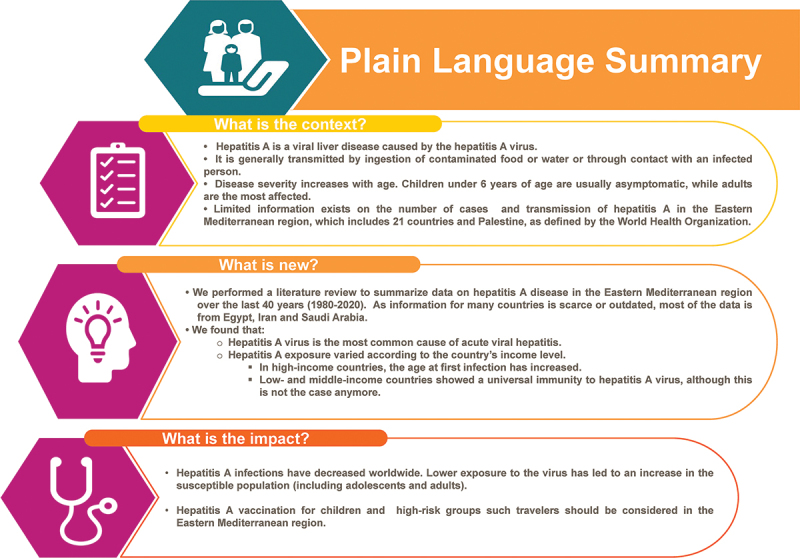
Plain language summary.

There is limited information on the epidemiology of HAV disease in EMR countries, specifically in relation to shifts of HAV endemicity.^16,17^ This review aims to explore HAV epidemiology by collecting and summarizing the serological data from the EMR region. The review highlights the importance of the EMR as a globalized hub for travel, migration, and food trade to bring awareness toward the probability of future global outbreaks of HAV disease (Figure 1).

## Methods

A comprehensive review utilizing a systematic approach was performed to identify published literature on HAV incidence and seroprevalence in the WHO-EMR^13^ covering 22 countries according to the preferred reporting items for systematic reviews and meta-analyses (PRISMA) guidelines.^18^ According to these guidelines, we defined search sources, search strategy, the inclusion, and exclusion criteria to identify and select relevant publications, and the scope of data extraction prior to the conduct of the review.

### Search sources and strategy

The search was conducted in MEDLINE (via PubMed) and complemented with a search of gray literature sources such as Ministry of Health (MoH) websites and reports from universities. We developed a broad search strategy using free-text terms (”HAV”; “COUNTRY NAME”) and medical subject heading (MeSH) terms linked by Boolean operators.

Searches were limited to a period of 40 years, i.e., from 1980 to July 2020. The lower limit of the period was considered appropriate by the authors as it allows to observe shifts in the burden of disease, if any. The countries of interest, based on the geographic scope of this review, were limited to the WHO-EMR covering 22 countries. Searches were conducted in both English and the local language of each included country (Table 1).

**Table 1.** Inclusion and exclusion criteria.

	Inclusion criteria	Exclusion criteria
Population	Hepatitis A disease (not limited to risk groups or specific ages)	Populations with chronic diseases or underlying comorbidities that are not representative of the general population
Intervention	Not restricted by intervention	N.A.
Comparator	Not restricted by comparator	N.A.
Outcome	Proportion of HAV among all acute viral hepatitis (HAV IgM)	N.A.
	HAV seroprevalence (HAV IgG)	
Study design	Primary peer-reviewed research observational studies	Non-primary research
	Cohort studies	Systematic reviews
	Case-control studies	Meta-analyses
	Cross-sectional studies	Narrative reviews (without methods)
	Ecological studies	Predictions via modeling methods
	Outbreak investigations	Case reports
	Periodic surveys	Letter to editor
	Non-peer-reviewed research	Newspaper
	Reports from national and regional databases or websites	Editorial
		Comment
		Opinions
Limits		
Publication date	From 1980 onwards	All publications before 1980
Geographic scope	Afghanistan, Bahrain, Djibouti, Egypt, Islamic Republic of Iran, Republic of Iraq, Jordan, Kuwait, Lebanon, Libya, Morocco, Oman, Pakistan, Palestine, Qatar, Saudi Arabia, Somalia, Sudan, Syrian Arab Republic, Tunisia, United Arab Emirates, Yemen	All countries apart from those considered eligible
Language	English, French, Arabic	-

Note: HAV, hepatitis A virus; IgG, immunoglobulin G; IgM, immunoglobulin M; N.A, not applicable.

### Screening and selection

The identified publications were screened in two phases by two reviewers in an independent process using the inclusion and exclusion criteria listed in Table 1. The retrieved articles were initially screened by title and abstract for eligibility by one reviewer (AO, MK, YL, or OO) followed by a second step which included screening of the full text of articles using the eligibility criteria specified in Table 1. All discrepancies were discussed with an additional reviewer (SB).

Original research from non-interventional studies or from gray literature sources was included if it reported data on the occurrence of hepatitis A (defined as previous exposure to HAV confirmed by laboratory detection of HAV IgM) and seroprevalence of HAV (defined as previous exposure to HAV confirmed by laboratory detection of HAV IgG or total HAV immunoglobulin (Ig) in blood samples). Case reports and other publication formats such as commentaries, editorials, and letters were excluded from this review. Reviews and meta-analyses were consulted with the intention to screen their reference lists for eligible articles.

### Data extraction and reporting

The information extracted from selected studies included study characteristics (year of publication, study design, main objective of the study and sample size), age group of the study population and case definition (e.g., laboratory confirmation methods). The occurrence of HAV (HAV cases expressed as a proportion of all acute viral hepatitis cases) and HAV seroprevalence (expressed as a percentage of patients with previous exposure to HAV measured according to the test kit specifications) were extracted and reported. When available, the same outcomes were reported and compared by age group, socioeconomic status, year, type of setting (rural versus urban), and acute viral hepatitis caused by other types (hepatitis B virus, hepatitis E virus, etc.).

## Results

### Included studies and their characteristics

**Figure 2.**
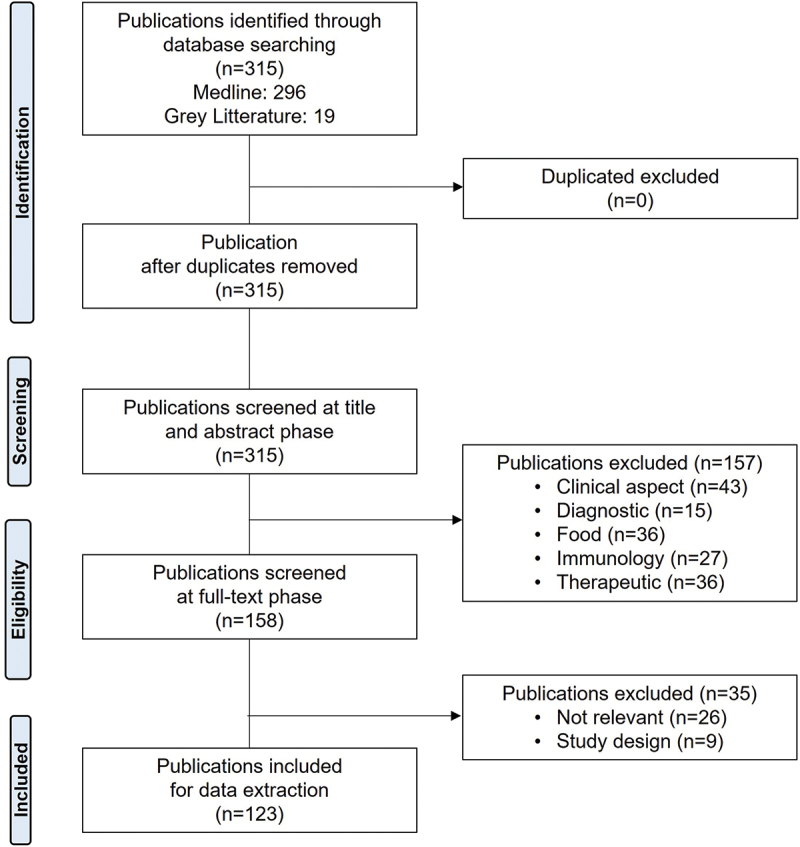
PRISMA flow diagram showing the study research and selection process.

Overall, the search yielded 315 publications (MEDLINE: *n* = 296; gray literature: *n* = 19). Of these, 157 were excluded at the title or abstract screening phase and 35 were further excluded after full-text review. Finally, a total of 123 publications for 22 countries in the EMR were included in the final review (Figure 2).

**Figure 3.**
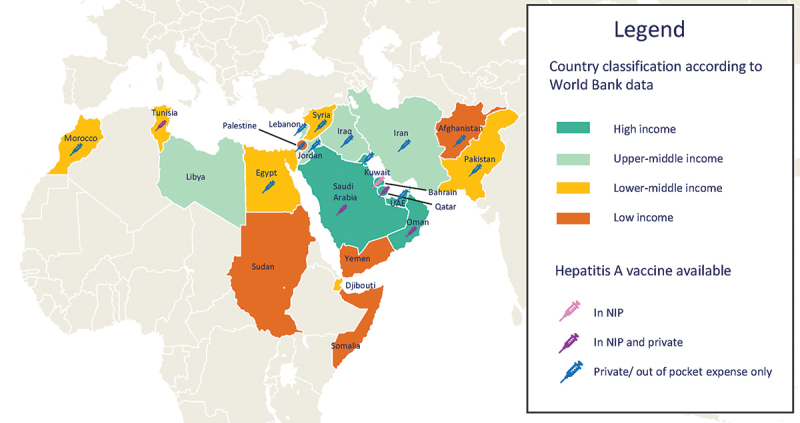
Classification of included countries by income level and hepatitis a vaccination status.

Notes: NIP, national immunization program; UAE: United Arab Emirates.

Among the 123 publications which provided data on hepatitis A disease for the 21 countries in the EMR and Palestine (Table 2), the distribution of publications by country was: Saudi Arabia (*n* = 30),^19–46^ followed by Iran (*n* = 28),^47–74^ Egypt (*n* = 19),^75–93^ Pakistan (*n* = 8),^94–101^ Lebanon (*n* = 6),^102–107^ Tunisia (*n* = 6),^108–113^ Iraq (*n* = 4),^114–117^ Kuwait (*n* = 3),^118–120^ Somalia (*n* = 3),^121–123^ Djibouti (*n* = 2),^124,125^ Jordan (*n* = 2),^126,127^ Syria (*n* = 2),^128,129^ UAE (*n* = 2),^130,131^ Yemen (*n* = 2),^132,133^ Afghanistan (*n* = 1),^134^ Libya (*n* = 1),^135^ Morocco (*n* = 1),^136^ Palestine (*n* = 1),^137^ Qatar (*n* = 1)^138^ and Sudan (*n* = 1) (Figure 3).^139^

**Table 2.** Demographic characteristics and HAV vaccination status of the 22 EMR countries.

COUNTRY	Geographic region	World bank classification (2017)^14^	GAVI eligibility (2017)^140^	HAV vaccination in NIP	Year of vaccination implementation	Availability of vaccine (private/public)	Recommendation status	Reimbursement
Afghanistan	Asia	LIC	Yes	No	N.A.	Private	-	No
Bahrain	Middle East	HIC	No	Yes	2012	Public	15 and 24 months High risk groups and travellers^141^	Reimbursed
Djibouti	Middle East	LMIC	NIP through GAVI support	No	-	-	No	-
Egypt	Africa	LMIC	No	No	-	Private	-	No
Iran, Islamic Republic Of	Asia	UMIC	No	No	-	Private	-	No
Iraq	Middle East	UMIC	No	High risk group	-	Private	-	No
Jordan	Middle East	UMIC	No	No	-	Private	-	No
Kuwait	Middle East	HIC	No	No	-	Private	Citizens born prior to 1990 and healthcare personnel^141^	No
Lebanon	Middle East	UMIC	No	No	-	Private	-	No
Libya	Africa	UMIC	No	No	-	-	No	-
Morocco	Africa	LMIC	No	No	-	Private	18 and 24 months	In private market: covered by insurance
Oman	Middle East	HIC	No	Yes	2020	Both	13 and 24 months	Reimbursed
Pakistan	Asia	LMIC	Yes	No	N.A.	Private	*	No
Palestine	Middle East	LIC	No	No	-	Private	18 and 24 months	No
Qatar	Middle East	HIC	No	Yes	2012	Both	12 and 18 months	Reimbursed
Saudi Arabia	Middle East	HIC	No	Yes	2008	Both	18 and 24 months	In private market: covered by insurance, Public: FoC for Saudi, non-Saudi and illegal immigrants
Somalia	Asia	LIC	Yes	No	-	-	-	-
Sudan, Republic of	Africa	LIC	Yes	No	-	-	-	No
Syrian Arab Republic	Middle East	LMIC	Yes	No	-	Private	-	No
Tunisia	Africa	LMIC	No	Yes	2019	Both	12 months and 6 years	In private market: covered by insurance, Public: FoC
United Arab Emirates	Middle East	HIC	No	No	-	Private	High risk groups and travelers^141^	No
Yemen	Middle East	LIC	Yes	No	-	-	-	-

*No local recommendations. Recommended by international bodies for HAV in case people are traveling to Pakistan.

FoC, free of charge; GAVI, the vaccine alliance; HIC, high-income countries; LIC, low-income countries; LMIC, low- middle- income countries; N.A., Not applicable; NIP, national immunization programs; UMIC, Upper-middle income countries; WHO-EMR, World Health Organization—Eastern Mediterranean Region.

No study dealing with HAV could be identified for Bahrain and Oman. Among the countries included in this review, childhood hepatitis A vaccination has been implemented in the national immunization programs (NIP) of Bahrain, Oman, Qatar, Saudi Arabia, and Tunisia and only for high-risk groups in Iraq (Table 2). In most countries, however, hepatitis A vaccination is available in the private market (Table 2 and Figure 3).

### Main findings from the review

#### Occurrence of HAV among acute viral hepatitis cases

A total of 41 studies provided data on HAV occurrence among all acute viral hepatitis cases. Overall, the proportion of HAV cases among acute viral hepatitis cases was large and ranged from 1.5% to 97.0% (Table 3). One study reported an increase in the proportion of HAV from 2001–2004 (40.2%) to 2014–2017 (89.7%); and reported a reduction in the proportion of patients infected with HAV before five years of age and an increase in the proportion of patients infected in an older age group.^89^ In patients with acute viral hepatitis, coinfection with hepatitis B, C, and E was documented in nine studies^83,86,87,91,92,98,116,120,133^ (Table 3).

**Table 3.** Occurrence of HAV among acute viral hepatitis cases (41 studies).

Studies by country	Data period, year(s)	Study population (number, age restrictions)	HAV, % (n)
**Djibouti**			
Coursaget et al., 1998^125^	1992–1993	111 pts, 2–65 y	33% (37)
**Egypt**			
Fouad et al., 2018^85^	2015–2017	268 pts, 1–18 y	97% (260)
Talaat et al., 2019^89^	2014–2017	9,321 pts, all ages	93.4% (7,806)
Hasan et al., 2016^86^	2007–2008	123 pts, 2–18 y	13.8% (17)
Eldin et al., 2010^84^	2007–2008	235 pts, 1–65 y	8.1% (19)
Meky et al., 2006^88^	2002–2005	47 community residents, 2–77 y	8.5% (4)
Talaat et al., 2010^90^	2001–2004	5,909 pts, all ages	28.5% (1,684)
Zakaria et al., 2007^92^	2001–2002 1983	200 pts, all ages 235 pts, all ages	34% (68) 2.1% (5)
Hyams et al., 1992^87^	1987–1988	73 outpatients, ≤13 y	41% (30)
Divizia et al., 1999^83^	1993	202 hospitalized pts, 1–73 y	10.4% (21)
Youssef et al., 2013^91^	n.r.	33 hospitalized children	33% (11)
Zaki Mel et al., 2008^93^	n.r.	162 children	34.1% (n.r)
Darwish et al., 1992^82^	n.r.	200 adult pts, 20–40 y	4.5% (9)
**Iran**			
Karimi et al., 2015^57^	2010	70 pts	68.6% (48)
**Iraq**			
Al-Naaimi et al., 2012^114^	2010–2011	2,629 pts, all ages	44.8% (1,206)
Turky et al., 2011^117^	2005–2006	2,975 pts, all ages	41% (1,219)
Marcus et al., 1993^115^	n.r.	107 pts, 1.5–65 y	40.2% (43)
Rassam et al., 1989^116^	n.r.	253 hospitalized pts, 3–65 y	15% (39)
**Kuwait**			
Al-Kandari et al., 1986^120^	1983–1984	1,788 pts, all ages	1.5% (26)
Al-Kandari et al., 1987^119^	1980–1984	52 pregnant pts, 15–44 y	11.5% (6)
**Lebanon**			
Shamma’a et al., 1984^106^	1980–1981	93 pts, >12 y	35.5% (33)
**Pakistan**			
Khan et al., 2011^99^	2007–2008	89 pts, all ages	6.1% (4)
Ahmed et al., 2010^97^	1987–2007	346 outpatients, all ages	3.5% (12)
Waheed-uz-Zaman et al., 2006^101^	2003–2004	626 pts, all ages	40.6% (252)
Syed et al., 2003^100^	1994–1999	658 pts, 11 y and over	64.4% (424)
Haider et al., 1994^98^	1991	93 hospitalized pts, all ages	5.4% (5)
**Qatar**			
Glynn et al., 1985^138^	1981	126 hospitalized pts, 13–52 y	5.5% (7)
**Saudi Arabia**			
Al-Tawfiq et al., 2008^32^	2000–2005	1,214 pts, 1–94 y	10% (120)
Memish et al., 2003^43^	1999–2001 • 1999: • 2000: • 2001:	3,490 pts, all ages • 1,194 pts • 1,039 pts • 1,257 pts	8.2% (286) • 6.7% (80) • 6.9% (72) • 10.7% (134)
Fathalla et al., 2000^40^	1987–1999	683 pediatric pts	65% (641)
Ayoola et al., 2001^37^	1997–1998	246 pts, all ages	37% (91)
Arif et al., 1995^34^	1993–1994	133 pts, all ages	38.3% (51)
Yohannan et al., 1990^46^	1987	47 pts, <12 y	72% (34)
Al-Majed et al., 1990^28^	n.r.	23 pts, all ages	82.6% (19)
Al-Knawy et al., 1997^27^	n.r.	132 hospitalized pts, >3 y	81.8% (108)
**Sudan**			
Hyams et al., 1991^139^	1987–1988	80 outpatients, <14 y	33.8% (27)
**Syria**			
Al-Azmeh et al., 1999^129^	1995–1998	193 pts, >12 y	53.9% (104)
**Tunisia**			
Neffatti et al., 2017^110^	2014–2015	92 pts, 1-62 y	21.7% (20)
Gharbi-Khelifi et al., 2012^112^	2006–2008	400 pts, 1-60 y	19.8% (79)
Hellera et al., 2014^113^	2004–2005	105 pts, 15–65 y	34.3% (36)
**Yemen**			
Gunaid et al., 1997^133^	n.r.	78 pts, ≥13 y	5.1% (4)

HAV, hepatitis A virus; n, number of study participants who were anti-HAV positive; n.r., not reported; pts, patients; y, years.

#### HAV seroprevalence

A total of 77 studies provided data on HAV seroprevalence. HAV seroprevalence ranged from 3% to 100%, depending on the age of the study population (Table 4). Overall, the EMR region has an intermediate level of HAV seroprevalence, and the data show a remarkable consistency. While seroprevalence studies from before the year 2000 showed nearly universal immunity among the general population in many countries of the EMR, after the year 2000, seroprevalence rates reveal that more adolescents and adults remain susceptible to HAV, although with significant variation within the region.

**Table 4.** Seroprevalence of HAV (77 studies).

Studies by country	Data period, year(s)	Study population (number, age restrictions)	HAV seroprevalence (IgG), % (n*)		
**Afghanistan**					
Carmoi et al., 2009^134^	2008	102 anicteric pts, 5–65 y	99% (101) • <15 y: 91.7% • ≥15 y: 100%		
**Djibouti**					
Fox et al., 1988^124^	1987	400 healthy adults	98.5% (394)		
**Egypt**					
El-Karaksy et al., 2008^77^ and El-Karaksy et al., 2006^78^	2004	101 children with chronic liver disease (CLD), <18 y	85.1% (86) • <5 y: 62.1% • ≥5 y: 94.4%		
Al-Aziz et al., 2008^75^	2002–2003	296 children with minor illnesses, 2.5–18 y	61.4% (181) • 2.5–6 y: 53.1% • 6–9 y: 56.4% • 9–18 y: 73.8%		
Salama et al., 2007^81^	2003–2004	426 children with minor medical problems,3–18 y	86.2% (367) <6 y: 64.3% 6–10 y: 85.3% 11–15 y: 90.3% >15 y: 90%		
Darwish et al., 1996^76^	1994	155 healthy community residents, 1–67 y	100% (155) • 1–3 y: 100% • ≤67 y: 100%		
Kamel et al., 1995^79^	1992	1,259 healthy community residents, all ages	97.2% (1224) • 0–4 y: 92.7% • 5–9 y: 97.8% • 10–14 y: 97.9% • 15–19 y: 97.5% • 20–24 y: 96.6% • 25–29 y: 97.9% • 30–34 y: 95.5% • 35–39 y: 100% • 40–44 y: 93.3% • 45–49 y: 100% • 50–54 y: 97.6% • 55–59 y: 93.3% • 60–64 y:100% • 65–69 y: 100% • >70 y: 100%		
Omar et al., 2000^80^	n.r.	228 community residents, preschool children	26.3% (60)		
**Iran**					
Mirzaei et al., 2016^60^	2014–2015	108 hemophilic pts, 4–85 y	77.8% (84)		
Hesamizadeh et al., 2016^53^	2014	559 volunteer blood donors, >18 y	70.7% (395) • 18–27 y: 26.7% • 28–37 y: 59.8% • 38–47 y: 91.2% • >47 y: 94.8%		
Hosseini Shokouh et al., 2015^74^	2012–2014	270, healthy medical students, 18–30 y	34.8% (94)		
Vasmehjani et al., 2015^73^	2012–2013	159, CLD pts, 21–68 y	79.2% (126) • 21–30 y: 28.6% • 31–40 y: 91.4% • 41–50 y: 93.9% • <50 y: 95%)		
Izadi et al., 2016^55^	2011–2013	1,554, healthy soldiers, 18–60 y	80.3% (1,248) • <20 y: 72.2% • 20–30 y: 79.1% • >30 y: 92.4%		
Farajzadegan et al., 2014^52^	2003–2013	11,857 cumulative population of 16 studies (systematic review), all ages	51%–66%		
Jahanbakhsh et al., 2018^56^	2012	569 homeless adults, 18–60 y	94.3% (nr) • <42 y: 90.3% • ≥42 y: 98.1%		
Asaei et al., 2015^48^	2011–2012	1,030, healthy individuals, 0.5–95 y	66.2% (682) • 6–15 y: 18.3% • 16–29 y 79.4% • 30–55 y: 94.3% • ≥56 y: 98.2%		
Bayani et al., 2013^50^	2011–2012	466 healthy healthcare workers	71% (330) • 20–29 y: 57.8% • 30–39 y: 77.1% • >40 y: 86.3%		
Rabiee et al., 2013^63^	2011	1,813, healthy university students	39.8% (722)		
Shoaei et al., 2012^69^	2010–2011	117, chronic hepatitis C pts	94.9% (111) • ≤30 y: 93.1% • 31–40 y: 93.3% • 41–50 y: 100% • 51–60 y: 100% • ≥61 y: 100%		
Vakili et al., 2014^72^	2010	1,028, healthy 1^st^ year medical students,17-27 y	68.5% (704)		
Saffar et al., 2012^67^	2010	984, community residents, 1–30 y	19.2% (189) • 1–2.9 y: 5.7% • 3–6.9 y: 9.1% • 7–10.9 y: 20.4% • 11–17.9 y: 34.8% • 18–30 y: 68.4%		
Mostafavi et al., 2016^62^ and Hoseini et al., 2016^54^	2009–2010	2,494, national health survey participants,10–18 y	50.4%–78.8% across provinces 64% (1,597)		
Sofian et al., 2010^70^	2009	1,065, pediatric hospital pts, 0.5–20 y	61.6% • 0.5–1.9 y: 61.5% • 2–5.9 y: 51.7% • 6–10.9 y: 52.9% • 11–15.9 y: 65.2% • 16–20 y: 85.0%		
Taghavi et al., 2011^71^	2008–2009	1,050, pre-marriage lab analysis, 15–63 y	88.2% (927) • <20 y: 79.3% • 20–30 y: 91.3% • >30 y: 99%		
Ramezani et al., 2011^64^	2008	351, blood donors, 17–60 y	94.9% (333)		
Saneian et al., 2014^68^	2007	361, healthy medical students	75.3% (272)		
Alian et al., 2011^47^	2007	1,034, community residents, 1–25 y	38.9% (402) • 1–5 y: 8.9% • 5–15 y: 15.8% • 15–25 y: 64.3%		
Mohebbi et al., 2012^61^	2006–2007	551, community residents, 1–83 y	90.0% (496) • <30 y: 85.7% • 30–60 y: 90.7% • >60 y: 93.9%		
Merat et al., 2010^59^	2006	1,869, community residents, 18–65 y	86%		
Davoudi et al., 2010^51^	2005–2006	247 HIV+, 5–74 y	96.3% (238)		
Ataei et al., 2008^49^	2006	816, community residents, >6 y	8.3%		
Roushan et al., 2007^65^	2004–2005	392, HBsAg+ pts, 10–70 y	82.1% (332) • 10–19 y: 59.4% • 20–29 y: 89.8% • >29 y: 97.5%		
Mehr et al., 2004^58^	2002	1,018, children in pediatric hospital, 0.5–15 y	22.3% (227)		
Saberifiroozi et al., 2005^66^	n.r.	204, pts in liver clinic, adults	98% (200)		
**Jordan**					
Hayajneh et al., 2015^127^	2008	3,066, community residents, 0–85 y	51% • ≤1 y: 24% • 1–2 y: 26% • 2–4 y: 32% • 5–9 y: 44% • 10–14 y: 63% • 15–19 y: 78% • >20 y: 94%		
**Kuwait**					
Alkhalidi et al., 2009^118^	2003–2004	2,851, healthy adults	28.6% (816) • 18–27 y: 24.2% • 28–40 y: 51% • 41–60 y: 56.5%		
**Lebanon**					
Melhem et al., 2015^104^	2012–2013	283, blood donors	72% • 19–29 y: 60% • 30–39 y: 77% • 40–49 y: 94% • 50–59 y: 91%		
Bizri et al., 2006^102^	1999–2000	902, school children, 14–18 y	71.3% (643)		
Kalaajieh et al., 2000^103^	1996–1998	740, pediatric clinic pts, 0.5–15 y	29.3% (217) • 0.5–6 y: 14.7–21% • 7–15 y: 37.6–40.1%		
Sacy et al., 2005^105^	1999–2000	606, healthy volunteers visiting or working in four hospitals, 1–30 y	43.2% (262) • 1–5 y: 10.5% • 6–10 y: 27.7% • 11–15 y: 57.4% • 16–20 y: 70.1% • 21–30 y: 78.1%		
Shamma’a et al., 1982^107^	n.r.	772, mixed sample of pts	• Lebanese adults: 97.7% (474/485) • Pediatric group: 79.5% (136/171) • Foreign adults: 38.8% (45/116)		
**Libya**					
Gebreel et al., 1983^135^	1979–1981	400, school children, 3–18 y	60%–100%		
**Morocco**					
Bouskraoui et al., 2009^136^	2005–2006	150, children, 0.5-14 y	51% • ≤6 y: 45.2% • >6–14 y: 70.3%		
**Palestine**					
Yassin et al., 2001^137^	n.r.	396, school children, 6–14 y	93.7% • 6 y: 87.8% • 14 y: 97.5%		
**Pakistan**					
Aziz et al., 2007^95^	2002–2004	380, children from squatter settlements,<18 y	≥14 y: 100%		
Agboatwalla et al., 1994^94^	1990–1991	258, healthy children (239) and adults (19)	55.8% (144) • <5 y: 41% (98/239) • 30–50 y: 100% (19/19)		
Hamid et al., 2002^96^	n.r.	233, adult outpatients with CLD	• 97.8% (228)		
**Saudi Arabia**					
Alshabanat et al., 2013^31^	2006–2010	44,679, viral hepatitis pts, all ages	17% (7,566)		
Al-Faleh et al., 2008^21^	2007–2008	1,357, school children, 16–18 y	18.6% (253)		
El-Gilany et al., 2010^39^	2006–2007	950, children attending regular vaccination schedule, 1–6 y	33.8% (321)		
Almuneff, et al., 2006^29^	2001–2005	4,006, healthcare workers	67%		
Almuneef et al., 2006^30^	2005	2,399, all ages	28.9% (694) • <8 y: 7.1% • 8–11 y: 14.5% • 12–15 y: 30.6% • ≥16 y 52%		
Jaber, 2006^41^	2004	527, aged 4–14 y	•28.7%		
Al-Ghamdi et al., 2004^26^	2003	650, children − 1st year primary school	8.2% (53)		
Fathalla et al., 2000^40^	1987–1999	11,674, healthy children and adults (18–50 y)	86% (10,029) • children: 65% • adults: 78.8% Detailed in children: • <6 y: 3% • 6–<8 y: 62% • 8–<10 y: 71% • 10–<12 y: 83% • 12–<18 y: 93%		
Al-Faleh et al., 1999^24^	1997	5,355, community residents, children 1–12 y	25% (1,331) • 1–2 y: 16% • 3–4 y: 22% • 5–6 y: 25% • 7–8 y: 29% • 9–10 y: 34% • 11–12 y: 34%		
Khalil et al., 1998^42^	1995–1996	592, children in regular appointments or inpatient care, <16 y	30.2% (179) • 0.5–2 y: 12.5% • 3–4 y: 14.7% • 5–6 y: 20.3% • 7–8 y: 40.4% • 9–10 y: 32% • 11–12 y: 44.3% • 13–15 y: 48.6%		
Al Rashed, 1997^23^	1989	4,375, community residents, children, 1–10 y	52.4%		
Ashraf et al., 1986^35^	1985	55, hemodialysis pts, all ages	100%		
Ashraf et al., 1986^36^	1984–1985	395, healthy blood donors or minor illness pts, all ages	89% (353) • <0.5 y 65.5% • 0.5–2 y: 60% • 3–5 y: 83.3% • 6–12 y: 97.8% • >13 y: 100%		
Babaeer et al., 2011^38^	n.r.	1,050, pts, >2 y	33.1% (348) • 2–5 y: 17% • 6–9 y: 21.1% • 10–14 y: 28.8% • 15–19 y: 27.2% • 20–24 y: 34.3% • 25–29 y: 38.2% • 30–34 y: 47.7% • >35 y: 49.2%		
Al-Faleh et al., 2010^22^	n.r.	1,157, school children, 16–18 y	16.4% (190)		
Arif, 1996^33^	n.r.	1,418, community residents, all ages	68.0% (964) 1–12 y: Riyadh, 24.7%; Gizan, 35.1% >13 y: Riyadh, 77.6%; Gizan, 90.9%		
Ramia, 1986^45^	n.r.	1,015, Riyadh residents, all ages	82.5% (837) • <1 y: 67.9% • 1–4 y: 38.6% • 5–9 y: 61.3% • 10–15 y: 81.5% • 16–19 y: 83.5% • 20–29 y: 91% • 30–39 y: 93.5% • ≥40 y: 95%		
**Somalia**					
Hassan-Kadle et al., 2018^121^	[4 studies published from 1984 to1994]	Participants in the 4 studies, all ages	90.2% • <1 y: 61.5% • 1–10 y: 91.9% • 11–19 y: 96.3% • 20–39 y: 91.3% • ≥40 y: 87%		
Bile et al., 1992^122^	n.r.	672, children in 2 residential institutions, <18 y	By institution: • 96% (Shebeli) • 59% (Societe Organization Sociale)		
Mohamud et al., 1992^123^	n.r.	593, 0-83 y	•90%		
**Syria**					
Antaki et al., 2000^128^	n.r.	849, all ages	89% (754) • 1–5 y: 50% • 6–10 y: 81% • 11–15 y: 95% • 16–20 y: 94% • 21–30 y: 97% • 31–40 y: 98% • 41–50 y: 100%		
**Tunisia**					
Neffatti et al., 2017^110^	2014–2015	216 pregnant women, 19-46 y	98.6% (212)		
Louati et al., 2009^109^	2000 & 2007	376 blood donors, 18–30 y		2000	2007
			18–20 y	91.9%	80.6%
			21–25 y	93.7%	84.9%
			>26 y	99.2%	92.1%
			Total	94.9%	85.9%
Rezig et al., 2008^111^	n.r.	2,482, community residents, children and young adults	87.9% (2,180) • 5- <10 y: 83.9% • 10–15: 90.5% • 16–25 y: 91.9%		
Letaief et al., 2005^108^	2002	2,400, school children, 5–20 y	60% • 5–10 y: 44% • 10–15 y: 58% • 15–20 y: 83%		
**United Arab Emirates**					
Sheek-Hussein et al., 2012^130^	2011–2012	261, healthy medical students	21%		
Sharar et al., 2008^131^	2004–2005	367 children attending hospital, 1–12 y	20.1% (74) • 1–6 y: 10.2% • 6–12 y: 31.5%		
**Yemen**					
Bawazir et al., 2010^132^	2005	538, pts attending hospitals, all ages	86.6% (466) • 0–1 y: 53% • <18 y: 80.8% • ≥18 y: 98.8%		

CLD, chronic liver disease; HAV, hepatitis A virus; HBsAg, surface antigen of the hepatitis B virus; HIV, human immunodeficiency virus; IgG, immunoglobulin G; n, the number of study participants who were anti-HAV positive (* if available); n.r. not reported; pts, patients; y, year(s).

Main observations from the different countries are summarized in Table 4. In Afghanistan, a high seroprevalence (99%) was documented; HAV seroprevalence was higher among individuals >15 years of age compared to those <15 years of age (100% versus 91.7%).^134^ A study from 1987, in Djibouti, reported a prevalence of 98.5%.^124^ Seroprevalence surveys conducted in Egypt in the 1990s^76,79^ generally depicted a high immunity rate among children ≤5 years of age with 97.2–100% anti-HAV antibody prevalence. Studies from Egypt in the 2000s showed that 61.4%^75^ to 86.2%^77,81^ of children ≤6 years of age had immunity, and that 85.1% of patients with chronic liver disease had immunity.^77,78^ Studies from Iran indicate that most children and teenagers are susceptible to hepatitis A infection^47,48,65,67,70^ (Table 4). One study from Jordan provides strong evidence for continuous transition of HAV epidemiology toward intermediate endemicity, with increasing proportions of susceptible adolescents and adults.^126,127^ A study conducted in Lebanon in the early 1980s highlighted that 79.5% of children had anti-HAV antibodies.^107^ Studies conducted in 1999 and 2000 showed that more than half of teenagers had immunity, and about 20% of young adults remained susceptible to infection.^102–105^ Studies in Pakistan in the 1980s, 1990s, and 2000s indicate that more than half of children acquire immunity by their preschool years and nearly all adolescents and adults are immune.^94–96^ Earlier seroprevalence surveys conducted in Saudi Arabia generally reported high proportions of children and teenagers with acquired immunity,^23,36,40,45^ but noted lower seroprevalence in urban areas.^33,42^ In the same population, studies after the 2000s generally report lower immunity levels^21,30,41^ (Table 4). Studies from Kuwait,^118^ Tunisia,^108^ and the United Arab Emirates^131^ conducted in the 2000s show 10.2 to 31.5%^131^ HAV seroprevalence in children, and immunity in only 21% of young adults.^130^ In Morocco, the high overall HAV prevalence reported in 2005–2006 in children confirms that Morocco is an intermediately endemic area for HAV infection and is entering a transitional phase.^136^ Infection rates in children were high in other countries, such as in Libya,^135^ Yemen,^132^ Somalia,^121,122^ Syria,^128^ Tunisia^111^ and in some special populations, such as those living in Palestine.^137^

#### Temporal trends in HAV seroprevalence

Five studies reported HAV seroprevalence over time.^21,24,42,92,109^ These studies reveal that the HAV frequency rate is decreasing over time; this reduced force of infection has significantly increased the average age at infection. One study documented an increase in HAV occurrence in a large Egyptian hospital from 2.1% (1983) to 34% (2002); this is likely caused by delayed initial exposure to HAV resulting in symptomatic cases at older ages.^92^ Most of these cases occurred in older age groups, with only 20 (29%) of 68 infected patients being younger than five years, compared to 80% in 1983, and 22 (32%) of 68 patients above 9 years of age compared with 1 (20%) of 5 patients in 1983.^92^

#### Socioeconomic aspects of HAV seroprevalence

HAV seroprevalence data by area of residence was reported in 10 studies. Overall, a higher seroprevalence of HAV was generally reported among individuals residing in rural areas compared to urban areas, likely due to limited access to improved water sources and to sanitation facilities.^23,26,47,52,55,59,60,62,89,90^ Four studies reported data on HAV seroprevalence by socioeconomic status;^21,23,75,81^ collectively the data shows that individuals or families from low-income households (36.8 to 87.7%) had higher HAV seropositivity compared to individuals from middle- or high-income households (5.9 to 50.7%).

## Discussion

To our knowledge, this is the first comprehensive review of hepatitis A epidemiology in the EMR. We expect the findings of this review to help raise awareness and inform the development of appropriate interventional strategies to manage the evolving epidemiological situation in the region as well as globally. In recent decades, HAV seroprevalence has been declining in most parts of the world, mainly due to improvement in socioeconomic status, better access to clean water, sanitation, and in some cases, to active immunization. In the EMR, HAV seroprevalence rates are generally high with recent evidence indicating a delay of viral exposure into adulthood in most countries of the region.^140^ This change leaves older children, adolescents, and adults more likely to develop overt disease. Similar observations have been made in other developing countries in Asia (India, Thailand, and Taiwan),^141^ Latin America (Argentina, Brazil, Chile, Dominican Republic, Mexico, and Venezuela)^142^ including a recent comprehensive review on all Latin American countries,^143^ and Africa (South Africa).^144^ Given that the severity of HAV symptoms increases with age,^3^ it may be appropriate for the EMR countries with a high proportion of susceptible older children and adults to consider implementing HAV vaccination programs. These programs could target certain populations such as young children, and simultaneously could foster improvements in access to clean water, sanitation, and hygiene in the region.^2^

Considering the evolving situation with regard to international trade (specifically food and travel) and rising conflict in the region, the epidemiological context in the EMR is expected to have consequences for global public health. Measures such as immunization of risk groups like travelers and food handlers, and the creation of a common standard for the health, reception, and reporting of asylum seekers and refugees from this region should be considered. Advances in modern transportation and global accessibility have boosted the travel industry in the region. In Europe, travel continues to cause both imported cases and secondary transmission.^145^ Travel to and from countries with high or intermediate HAV endemicity is a risk factor for infection in residents of countries with low HAV endemicity, such as countries in Europe and North America. Individuals may be exposed to HAV during their travels and thus may transmit the imported infection within their communities, leading to subsequent outbreaks.^140^ GeoSentinel, the global surveillance network of the International Society of Travel Medicine reported 120 cases of hepatitis A among 737 international travelers to India, Egypt, Morocco and Mexico, between 2007 and 2011.^146^ Another study reported that 80 cases of HAV infection were diagnosed among European travelers returning from Egypt.^147^ Two concurrent travel-related HAV clusters were detected in eight European countries after travel to Morocco.^148^

EMR countries have undergone rapid urbanization and changes in lifestyle and consumer demands. These changes have had a profound effect on the production, supply, availability, and consumption of food.^149^ In the last few decades, international food trade from the EMR has accelerated but the recent coronavirus disease 2019 (COVID-19) pandemic has, at least temporarily, brought this to a standstill. Notwithstanding the effects of COVID-19 on global travel and trade, risks of HAV contaminated food remain high, with the WHO Foodborne Disease Burden Epidemiology Reference Group estimating that more than 90,000 deaths occurred worldwide due to acute viral hepatitis in 2010. Nearly 30,000 of those deaths could be due to foodborne transmission of HAV.^150^ The risk is elevated when food products are imported from high and intermediate HAV endemic countries or from countries with poor food processing practices.^149^ Furthermore, the HAV capsid has a highly stable molecular structure which allows it to persist in certain types of foods for extended periods of time and withstand common food processing practices.^151^ The European Union has reported two HAV infection outbreaks in 2013 due to frozen strawberries imported from Egypt and Morocco,^152^ and imported pomegranate seeds from Egypt have been traced as the source of an HAV infection outbreak in British Columbia, Canada, in 2012.^153^

Some areas in the EMR (*i.e*., Iraq, Iran, Syria, Palestine, and Yemen) are at the center of turmoil, with conflicts having a significant impact in these countries and beyond the region. The economic and health situation in these countries continues to worsen.^154^ Regional instability leads to difficulties in addressing public health issues while migratory movements are continuously being reported. One of the ramifications of migration from areas of conflict is the resurgence of infectious diseases such as hepatitis A, especially in low-endemic countries. This could possibly be driven by the influx of refugees and their settlement in underserved camps. Poor sanitation, hygiene, and inadequate supply of clean food and water in refugee camps are likely contributors to the rapid spread of HAV. A HAV outbreak was reported among Syrian refugees residing in hosting camps in Greece in 2016.^155^ A 45% increase in HAV cases among asylum seekers was reported in Germany in 2015–2016.^156^ In 2015, asylum applications in Europe amounted to approximately 1.35 million—a record since data collection began in 2008 and more than twice the number of applications than in 2014.^157^ While the COVID-19 pandemic may have slowed this trend due to restrictions affecting global travel and trade,^158^ careful monitoring of the situation and timely action to mitigate the risks of hepatitis A outbreaks are warranted.

There are some limitations of this review which are worth noting in the interpretation of the overall findings. A time limit was applied to the searches to identify publications beginning from 1980 onwards. This was considered appropriate by the authors to notice any shift in the burden of disease. More than half of the eligible studies identified in this review are from three countries (Egypt, Iran, and Saudi Arabia). Therefore, generalizability is limited to the countries from which most studies were reported and should not be extended to countries with very poor data representation, *i.e*., those with a few relevant studies or none at all. There is also a lack of consistency in study designs and age groups reported across the studies which prevents direct comparisons. This is compounded by the fact that the region is diverse with different income levels and healthcare infrastructure. Another factor that limits comparison is the different time periods considered within the studies. Finally, the data reported in this review was collected prior to COVID-19 and as such it does not reflect the travel and trade restrictions imposed on the countries in the EMR during the years 2020 and 2021. Due to these reasons, the overall findings should be interpreted with caution.

## Conclusion

In the EMR, hepatitis A remains a significant cause of acute viral hepatitis. While the populations in low-income countries show universal immunity to HAV, the middle- and high-income countries report increasing numbers of susceptible older children, adolescents, and adults which co-exist in rapidly developing societies. Given this shift in endemicity, it is expected that most of the countries in this region would experience a transition in HAV endemicity in the next decades, the consequence of which will be a higher burden of disease as the population ages, and the occurrence of outbreaks. The public health value of childhood vaccination against hepatitis A and of vaccinating only high-risk groups such as those traveling from and to the region should be assessed within this changing epidemiological context in the EMR.
